# The G-protein Coupled Receptor GPR8 Regulates Secondary Metabolism in *Trichoderma reesei*

**DOI:** 10.3389/fbioe.2020.558996

**Published:** 2020-11-05

**Authors:** Wolfgang Hinterdobler, Sabrina Beier, Alberto Alonso Monroy, Harald Berger, Christoph Dattenböck, Monika Schmoll

**Affiliations:** ^1^Center for Health & Bioresources, Bioresources, AIT Austrian Institute of Technology, Tulln, Austria; ^2^Symbiocyte, Tulln, Austria

**Keywords:** *Trichoderma reesei*, SOR cluster, secondary metabolism, heterotrimeric G-protein pathway, light response, chemosensing, cellulase, chemical communication

## Abstract

Changing environmental conditions are of utmost importance for regulation of secondary metabolism in fungi. Different environmental cues including the carbon source, light and the presence of a mating partner can lead to altered production of compounds. Thereby, the heterotrimeric G-protein pathway is of major importance for sensing and adjustment of gene regulation. Regulation of secondary metabolism is crucial in the biotechnological workhorse *Trichoderma reesei* for knowledge-based adjustment in industrial fermentations, but also with respect to the potential use as a host for heterologous compound production. We investigated the function of the class VII G-protein coupled receptor (GPCR) gene *gpr8* that is localized in the vicinity of the SOR cluster, which is responsible for biosynthesis of sorbicillinoids. GPR8 positively impacts regulation of the genes in this cluster in darkness. Accordingly, abundance of trichodimerol and dihydrotrichotetronine as well as other secondary metabolites is decreased in the deletion mutant. Transcriptome analysis moreover showed the major role of GPR8 being exerted in darkness with a considerable influence on regulation of secondary metabolism. Genes regulated in Δ*gpr8* overlap with those regulated directly or indirectly by the transcription factor YPR2, especially concerning genes related to secondary metabolism. The predicted FAD/FMN containing dehydrogenase gene *sor7*, one of the positive targets of the cascade triggered by GPR8, has a positive effect on secondary metabolite production, but also cellulase gene expression. Hence SOR7 has some overlapping, but also additional functions compared to GPR8. The G-protein coupled receptor GPR8 exerts a light dependent impact on secondary metabolism, which is in part mediated by the transcription factor YPR2 and the function of SOR7. Hence, *T. reesei* may apply GPR8 to adjust production of secondary metabolites and hence chemical communication to signals from the environment.

## Introduction

The ascomycete *Trichoderma reesei* (syn. *Hypocrea jecorina*) is one of the most prolific producers of plant cell wall degrading enzymes in industry. Therefore it has become a model organism for regulation of cellulase gene expression and plant cell wall degradation in general (Glass et al., 2013; Schmoll et al., 2016). Thereby, *T. reesei* regulates its enzyme production in response to its environment, most importantly depending on the amount and nature of the carbon source in its surroundings (Bazafkan et al., 2014). Regulation of plant cell wall degrading enzymes is governed by a complex network of transcription factors and regulators (Benocci et al., 2017). As the production of enzymes is an energy demanding process, the fungus only produces them when required. If easily metabolizable carbon sources like glucose are available, enzyme expression is repressed by carbon catabolite repression, which is triggered by the transcription factor CRE1 (Kiesenhofer et al., 2016; Adnan et al., 2017). Additionally, enzyme production is regulated by light in *T. reesei* on different carbon sources (Stappler et al., 2017a; Schmoll, 2018), resulting in strongly decreased cellulase levels in light compared to darkness (Stappler et al., 2017b).

The heterotrimeric G-protein pathway is among the most prominent signaling pathways in fungi (Li et al., 2007; Gruber et al., 2013). *T. reesei* has 57 G-protein coupled receptors, three G-alpha subunits and one G-beta and G-gamma subunit each (Gruber et al., 2013; Schmoll et al., 2016). The relevance of this pathway for sexual development (Tisch et al., 2011b; Seibel et al., 2012) and for cellulase gene expression has been studied in considerable detail (Schmoll, 2018), while other physiological functions of this pathway are less well studied in *T. reesei*. The two G-protein alpha subunits GNA1 and GNA3 both influence cellulase gene expression in a light dependent way on cellulose (Schmoll et al., 2009; Seibel et al., 2009), but constitutive activation does not lead to inducer independent cellulase production. Also the G-beta and gamma subunits GNB1 and GNG1 impact cellulase regulation (Tisch et al., 2011b). Only recently it was shown that the class XIII G-protein coupled receptor CSG1 regulates cellulase gene expression and hence secreted cellulase activity at the post-transcriptional level, while transcript abundance of cellulase genes remains largely at wild type levels (Stappler et al., 2017a).

Secondary metabolism in *T. reesei* has not been investigated in detail previously. As a biotechnological workhorse, *T. reesei* is not known as a producer of harmful toxins (Nevalainen et al., 1994; Frisvad et al., 2018). Only recently this topic received attention, which was mainly focused on the function of a gene cluster on chromosome 5 (Marie-Nelly et al., 2014). This cluster comprises two polyketide synthases and two transcription factors (Derntl et al., 2016; Monroy et al., 2017). Due to the biosynthesis of sorbicillin compounds by the enzymes in this cluster, it was termed SOR cluster (Monroy et al., 2017). The cluster was first described in *Penicillium chrysogenum* (Salo et al., 2016; Guzman-Chavez et al., 2017). Interestingly, this cluster was reported to be subject to birth and death evolution, which involved lateral gene transfer (LGT) and vertical transfer and is under strong selective pressure in the *Trichoderma* section Longibrachiatum (Druzhinina et al., 2016b). Accordingly, this cluster is not present in Sordariomycetes closely related to *Trichoderma*. The genes in this cluster are subject to regulation by the carbon catabolite repressor CRE1 in a light dependent manner upon growth on cellulose, which draws a connection between secondary metabolism and enzyme expression (Monroy et al., 2017). In agreement with this finding, YPR2, one of the transcription factors within the SOR cluster, impacts both carbon and secondary metabolism (Hitzenhammer et al., 2019). Also in a random mutant, increase in sorbicillinoid production accompanied with a decrease in cellulase production was observed, although the regulatory basis for this effect was not investigated (Li et al., 2018). Investigations of the function of YPR2 upon growth on glucose further support a carbon source dependent role of this transcription factor (Derntl et al., 2016). The biological relevance of the detected sorbicillin compounds requires more detailed studies. Recent reports suggest a function in pathogen defense, albeit a limited one (Derntl et al., 2017a).

As an impact of the SOR cluster and the sorbicillin compounds biosynthesized by its gene products on diverse physiological functions in *T. reesei* became obvious from previous reports, we were interested in the relevance of signal sensors potentially influencing regulation of the SOR cluster and their roles in growth and development. In the genomic area surrounding the SOR cluster, we found a gene encoding a class VII G-protein coupled receptor which is regulated by CRE1 in darkness as shown for other SOR cluster genes. Here we show that this G-protein coupled receptor indeed impacts secondary metabolism upon growth on cellulose, but not glucose in *T. reesei* and that its downstream pathway in part overlaps with that of YPR2.

## Materials and Methods

### Strains and Cultivation Conditions

The *T. reesei* strains QM6a (Martinez et al., 2008) and QM6aΔ*ku80* were used throughout this study along with the recombinant strains mentioned below. For testing phenotypes of mutants in sexual progeny, we used FF1 and FF2, the backcrossed, fertile derivatives of QM6a (Bazafkan et al., 2015; Tisch et al., 2017). For cultivation in liquid media, strains were revived from long term storage and then inoculated on malt extract agar (3% w/v) for growth in constant darkness for 14 days, which prevents interference of light pulses or circadian rhythms with transcriptome analysis. Mandels Andreotti minimal medium (Mandels and Andreotti, 1978) containing 1% (w/v) microcrystalline cellulose (Alfa Aesar, Karlsruhe, Germany) and 0.1% (w/v) peptone to induce germination was inoculated with 10^9^ conidia/L. Cultivation was done in constant light (1700 lux) or constant darkness at 28°C for 72 h at 200 rpm. Dark grown cultures were harvested with very low red safety light (darkroom lamp, Philips PF712E, red, 15 W). Sexual development of fertile derivatives was analyzed on malt extract agar (2% w/v) after 14 days at 22°C at daylight (12 h :12 h) as described previously (Seidl et al., 2009; Schmoll, 2013). Phenotypes were analyzed upon growth on Mandels Andreotti minimal medium supplemented with 2% agar and 1% (w/v) carboxymethyl cellulose (CMC) or 1% (w/v) glucose (Glc) after 72 h at 28°C in constant light with 1700 lux.

### Construction of Recombinant Strains

Vector construction for deletion of *gpr8* and *sor7* was performed by recombination cloning as described earlier (Schuster et al., 2012) using primers for flanking regions and PCR screening for *hph* marker constructs as described therein. QM6aΔ*ku80* was used as parental strain for deletions with the protoplast method and 50 μg/mL hygromycin B (Roth, Karlsruhe, Germany) as selection reagent (Gruber et al., 1990). Successful deletion was confirmed by PCR using primers binding within the deleted area. Copy number was determined as described previously (Tisch and Schmoll, 2013) and one copy of the deletion cassette was found for *gpr8* deletion and four copies for *sor7* deletion. DNA integrity for deletion screenings was confirmed by a parallel PCR using standard primers.

Phenotypes of deletion mutants were validated by crossing of the strains in the QM6a (MAT1-2) background with the female fertile strain FF1 (MAT1-1) to generate female fertile offspring of both mating types with the respective gene deletions. Presence of gene deletions as well as the mating type of ascospore-derived strains were analyzed by PCR. Presence of a functional copy of the *ham5* gene, which is required for female fertility and defective in QM6a (Linke et al., 2015; Tisch et al., 2017) was confirmed by high resolution melt curve analysis (HRM) in progeny.

The deletion of *sor7* in the wild-type QM6a caused a growth defect upon growth on carboxymethyl cellulose and glucose in light and in progeny of the crossing carrying the deletion (Supplementary Material, Data Sheet 1, Figure S1), indicating propagation of the mutation with the phenotype. Inherited deletion of *gpr8* led to a consistent phenotype of female sterility in MAT1-2 offspring with female fertile background (carrying a functional *ham5* copy). We conclude that the phenotypes of Δ*gpr8* and Δ*sor7* reflect the consequences of deletion of the respective genes.

### Isolation of Nucleic Acids and Analysis

Isolation of genomic DNA was performed by the phenol-chloroform method in order to obtain high quality DNA (Schmoll et al., 2004). For transformant screening we used the rapid minipreparation method for fungal DNA as described earlier (Liu et al., 2000).

For preparation of total RNA, fungal mycelia were harvested by filtration and frozen in liquid nitrogen. Isolation and quality control of fungal total RNA was done as described previously (Tisch et al., 2011a) and only high quality RNA was used for further analyses. RT-qPCR for selected genes of strains grown on 1% glucose in darkness (SOR-cluster genes and *cre1*) was performed as described previously (Tisch et al., 2011a; Bazafkan et al., 2017a) with *sar1* as reference gene as this gene emerged as most appropriate (Bazafkan et al., 2017a).

### Transcriptome Analysis and Bioinformatics

Preparation of cDNA from total RNA of three biological replicates and sequencing was performed at the core facility VetCORE (Vienna, Austria) on a HiSeq2000/SR50 machine. Mapping of high quality reads was done with bwa (Li and Durbin, 2009) using standard parameter settings and the genome data of JGI mycocosm^1^ (Martinez et al., 2008). Data processing was further performed with samtools (Li et al., 2009) and differential expression analysis as well as statistical evaluation were done using the limma package (Ritchie et al., 2015) as implemented in the software suite R.

For differential regulation a threshold of 2-fold regulation and a *p*-value of 0.01 was set. For gene annotations the comparative annotation list provided for *T. reesei*, *T. atroviride* and *T. virens* (Schmoll et al., 2016) as well as an annotation for *T. reesei* were used (Druzhinina et al., 2016a). Data are available under GenBank accession number GSE144529. Validation of results from transcriptome analysis was done by RTqPCR for two regulated genes (*pks11s*: 19.3-fold downregulation, *p*-value 0.00075 and *ypr2*: 7.9-fold downregulation, *p*-value 0.0053) as well as two un-regulated genes (*env1*: *p*-value 0.28 and *cbh1*: *p*-value 0.2; no significant regulation in both cases), confirming the reliability of the genome wide analysis. Primers used for this analysis were reported previously (Bazafkan et al., 2017b; Monroy et al., 2017).

The open source software HCE3.5 (Seo et al., 2006) was used for hierarchical clustering of gene expression patterns and co-regulation analysis with default settings applying the Poisson correlation coefficient as the similarity/distance measure. Functional category analysis was done with the FungiFun 2.0 online resource^2^ (Priebe et al., 2015). Phylogenetic analysis was done using MEGA4 as described previously (Bazafkan et al., 2015). Heatmaps of gene regulation of selected biosynthetic genes involved in secondary metabolism was created using the ClustVis online tool (Metsalu and Vilo, 2015).

### Biomass Determination and Specific Cellulase Activity

Determination of biomass formed by the fungal strains in the presence of insoluble cellulose was performed as described previously (Schmoll et al., 2005). Briefly, the fungal pellet containing biomass and cellulose was ground in liquid nitrogen, treated with 0.1 N NaOH and sonicated to break up cells. Protein content as liberated from mycelia was analyzed using the Bradford method reflecting amount of biomass relative to wild type.

Endo-1,4-β-D-glucanase activity was used as a measure of expression of cellulases in the supernatant of cultivations. Light- or dark grown cultures were analyzed using the azo-CMC-cellulose kit (S-ACMC-L, Megazyme, Wicklow, Ireland) according to the manufacturer’s instructions. For specific cellulase activity, enzyme activities in the supernatant were normalized to the produced biomass.

### Analysis of Secreted Metabolites

Culture filtrates harvested from cultivations under (Bazafkan et al., 2015; Tisch et al., 2017) as applied for transcriptome analysis were screened using standardized mass spectrometry as described previously (Sulyok et al., 2007). Targeted analysis for known compounds produced by fungi was performed essentially as described previously (Malachova et al., 2014; Monroy et al., 2017). Thereby more than 700 metabolites can be detected and calibration with serial dilutions of a multi analyte stock solution allows for reliable identification and quantification of secondary metabolites in the sample.

## Results

### A G-protein Coupled Receptor Located Close to the SOR Cluster

Screening the genome for potential regulators of sorbicillin production in *T. reesei* we found TR_53238 in the vicinity of the SOR cluster (Monroy et al., 2017). TR_53238 is located on scaffold 1: 413309-414991^3^, i.e., 67 kb upstream of the start of the SOR cluster (*pks11*/TR_73618). According to Marie-Nelly et al. (2014) the gene is encoded on chromosome 5 and has the designation TrE0638W in the most recent version of the genome by the Wang group (Li et al., 2017). Interestingly, the locus of TR_53238 is not syntenic in other fungi, not even in closely related *Trichoderma* spp. as the *pks* genes of the SOR cluster are located on different scaffolds in *T. atroviride, T. harzianum, A. nidulans, A. niger, P. chrysogenum*, and *B. bassiana*. Detailed evolutionary analysis indicated acquisition of the SOR cluster through lateral gene transfer (Druzhinina et al., 2016b). Hence localization of TR_53238 may have evolved differently in different fungi. TR_53238 encodes the only member of class VII (secretin-like) G-protein coupled receptors (GPCRs) in *T. reesei* and has homologs in *T. atroviride* and *T. virens* (Gruber et al., 2013; Schmoll et al., 2016). It has the typical composition of 7 transmembrane regions (Supplementary Material, Data Sheet 1, Figure S2) and is conserved in *Trichoderma* species as well as numerous plant pathogens such as *Fusarium* spp*., Botrytis cinerea, Sclerotinia sclerotiorum* etc., and also human pathogens like *Histoplasma capsulatum, Paracoccidioides brasiliensis* and *Aspergillus fumigatus* (Supplementary Material, Data Sheet 1, Figure S3). TR_53238 is related to *Aspergillus nidulans* gprM (Lafon et al., 2006), which is involved in glucose sensing and sexual development (Dos Reis et al., 2019) and its *N. crassa* homolog NCU03253/*gpr8* (Cabrera et al., 2015), that is indirectly regulated by the transcription factor ADV-1 (arrested development-1), which is necessary for transducing light signals and involved in controlling rhythms and cell fusion (Dekhang et al., 2017). *N. crassa* NCU03253 shows higher transcript levels upon growth on cellulose versus minimal medium and its transcript abundance increases over time with *Miscanthus* as carbon source (Tian et al., 2009). Therefore, this GPCR could exert functions in development, light response or cellulase regulation also in *T. reesei*. We designated the GPCR encoded by TR_53238 as GPR8.

### Gpr8 Is Regulated by Light and Associated With Metabolism

*Gpr8* is strongly down-regulated by light in QM6a (more than 10-fold) upon growth on cellulose (Hitzenhammer et al., 2019) and is also among the cAMP dependent targets of the photoreceptor ENV1 (Tisch et al., 2014). Moreover, *gpr8* is differentially regulated between different carbon sources (Stappler et al., 2017a). Comparative analysis showed up-regulation of *gpr8* under conditions of sexual development compared to growth on cellulose (>2-fold; *p*-value <0.005) in different *T. reesei* strains (Dattenböck et al., 2018). In order to gain first information on the function of GPR8 in *T. reesei*, we checked co-regulated genes under different conditions in publicly available datasets (Supplementary Material, Table 1). Genes co-regulated with *gpr8* upon growth on cellulose in wild type and photoreceptor mutants (Tisch and Schmoll, 2013) are strongly enriched in polysaccharide metabolism (*p*-value 1.26e-12). Upon growth on inducing and repressing carbon sources (Stappler et al., 2017a) co-regulated genes are enriched in functions associated with energy and respiration (*p*-value < 1.26 e-04) and particularly in protein synthesis (*p*-value 6.35e-55), but also in unfolded protein response (*p*-value 3.61e-03). Although not specifically enriched, co-regulated genes in both conditions comprised numerous genes involved in secondary metabolism. Coregulation under conditions related to development (Dattenböck et al., 2018) revealed significant enrichment of genes associated with metabolism (*p*-value 7.8 E-04) and in particular in secondary metabolism (*p*-value 2.4 E-03) (Supplementary Material, Table 1). Consequently, investigation of functions of GPR8 in enzyme production and secondary metabolism should reveal the physiological role of this receptor.

### GPR8 Exerts Its Major Function in Darkness

The proximity of *gpr8* to the SOR cluster as well as the co-regulation with genes involved in carbon and specifically polysaccharide metabolism is reminiscent of an interrelationship between regulation of carbon metabolism and secondary metabolism as shown recently (Monroy et al., 2017). Consequently, *gpr8* could be involved in balancing enzyme production with metabolite production in response to environmental conditions. Therefore we deleted *gpr8* in QM6a, which did not affect hyphal extension on plates, biomass formation on cellulose or sporulation. The effect of the *gpr8* deletion was validated by sexual development and progeny screening, which showed that the phenotype was propagated with the deletion (see above). We investigated the transcriptome of the strain lacking *gpr8* upon growth on cellulose in constant light and constant darkness. In order to validate our results, we tested whether regulation of crucial genes representative for cellulase expression or secondary metabolism in light and darkness are consistent between wild-type data in this experiment and previous data (Hitzenhammer et al., 2019). This was the case for *cbh1*, *cbh2*, *gpr8*, *grd1*, *ypr1*, and *ypr2*, which are down-regulated in light as well as for *env1*, *vel1* and *sub1*, which are up-regulated in light. Additionally we confirmed regulation patterns of selected genes by RTqPCR (see section “Materials and Methods”). Consequently, our experimental conditions were consistent with previous results and the data from our transcriptome analysis are valid.

**FIGURE 1:**
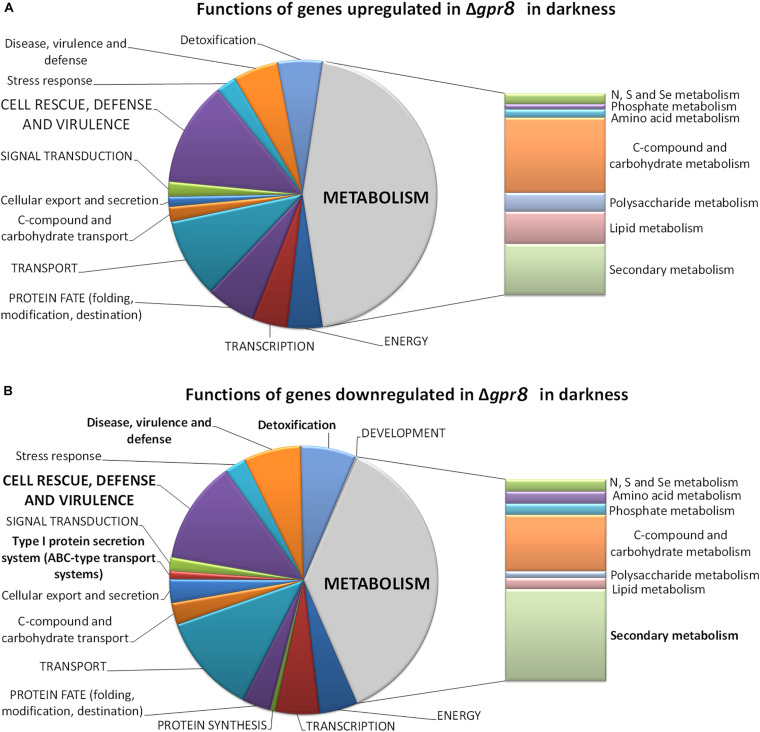
Functional category analysis of genes differentially regulated in Δ*gpr8* in darkness. **(A)** Functions of genes < 2-fold (*p*-value < 0.01) up-regulated in Δ*gpr8* in constant darkness upon growth on cellulose. **(B)** Functions of genes down-regulated in Δ*gpr8* in constant darkness upon growth on cellulose. Selected categories highly relevant for *T. reesei* physiology are shown.

In agreement with the considerably lower transcript abundance of *gpr8* in light compared to darkness (Hitzenhammer et al., 2019), we only found 38 genes regulated in Δ*gpr8* in light (Supplementary Material, Table 2). Besides a putative glycoside hydrolase, a carbohydrate esterase and a gene encoding a cytochrome P450 enzyme, only uncharacterized genes are in this gene group. In darkness, deletion of *gpr8* had a considerable impact on genes associated with metabolism, particularly secondary metabolism, but also C-compound and carbohydrate metabolism (Figure 1).

Among the genes up-regulated in Δ*gpr8* in darkness on cellulose we found significant enrichment of genes involved in secondary metabolism (*p*-value 1.36e-04), extracellular metabolism (*p*-value 3.10e-04) as well as extracellular protein degradation (*p*-value 1.53e-03) and interestingly also on oxidative stress response (*p*-value 3.27e-03) and catalase reaction (*p*-value 6.34e-05). The gene set of down-regulated genes showed significant enrichment for functions in metabolism (*p*-value 5.38e-04), particularly in genes associated with secondary metabolism (*p*-value 1.88e-17) indicating an important function of GPR8 in regulation of secondary metabolite production in response to the environment. Accordingly, functions in toxin transport (*p*-value 4.34e-12), type I protein secretion system (*p*-value 6.20e-05), disease, virulence and defense (*p*-value 3.07e-12) and detoxification (*p*-value 1.55e-11) are considerably enriched as well (Supplementary Material, Table 2).

### GPR8 Impacts Secondary Metabolism

We found 251 genes up-regulated in the mutant and 321 genes down-regulated upon growth on cellulose in darkness (Supplementary Material, Table 2). Among the genes up-regulated in darkness in Δ*gpr8* there are 18 CAZyme encoding genes including two glycoside hydrolase family (GH) 18 encoding genes and three family 10 carbohydrate esterase encoding genes as well as *cel61b*, *cip1* and *axe1*. Additionally, GPR8 negatively influences transcript levels of 2 genes encoding G-protein coupled receptors (including the pheromone receptor HPR2), four protease genes and seven genes encoding transcription factors as well as four transporter genes. Also three genes encoding cytochrome P450 proteins and the polyketide synthase gene *pks4g* are up-regulated in Δ*gpr8*. Up-regulation of three catalase encoding genes (*cat3*, *cat4*, and *cat7*) and one gene encoding a superoxide dismutase (*sod1*) suggests a function related to oxidative stress.

Analysis of genomic distribution of genes up-regulated in Δ*gpr8* revealed seven clusters (Supplementary Material, Table 2) of which cluster 2 and cluster 4 comprise CAZyme encoding genes.

Genes down-regulated due to the lack of GPR8 likely represent targets of the downstream cascade that respond to the signal transmitted by this G-protein coupled receptor. This gene set includes 15 CAZyme genes, including a polysaccharide lyase (TR_111245), a putative trehalase (TR_123226) and the alpha-galactosidase gene *agl1* and 10 transporter encoding genes. No cellulase or xylanase gene that would be expected to be regulated in case of a function in degradation of cellulose. Down-regulation of six G-protein coupled receptor genes, among them 5 PTH11 receptors, suggests an alteration of sensitivity to external signals in the absence of GPR8.

The hydrophobin coding gene *hfb1* is particularly strongly down-regulated in Δ*gpr8* (more than 14-fold).

**FIGURE 2:**
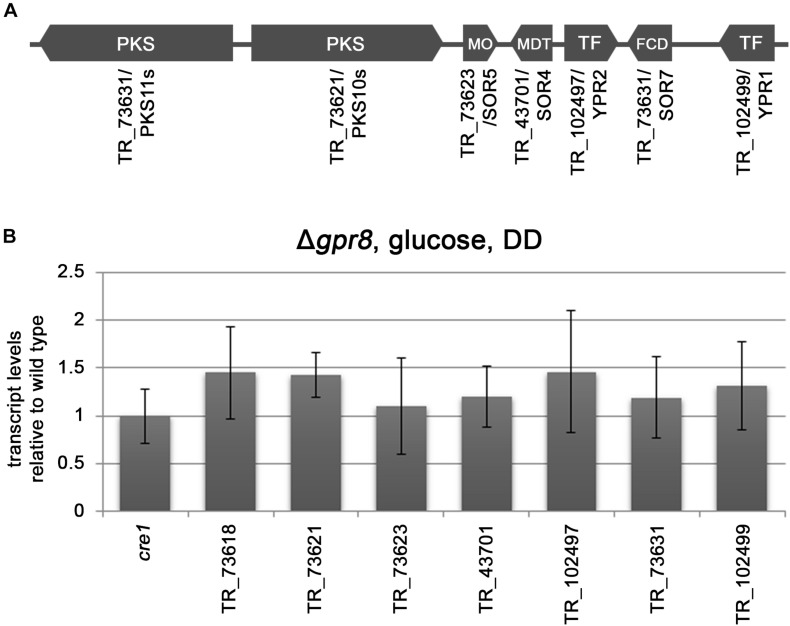
Transcript levels of *cre1* and SOR cluster related genes in Δ*gpr8* in darkness on glucose. **(A)** Schematic representation of the SOR cluster of *T. reesei* with protein IDs as listed on JGI (http://genome.jgi.doe.gov/Trire2/Trire2.home.html) along with gene designations. SOR7/TR_73631 was named SOR4 in Derntl et al. (2017a). **(B)** Transcript levels of *cre1* and SOR cluster assigned genes in Δ*gpr8* relative to wild type grown on 1% (w/v) glucose in darkness (DD). Transcriptional changes shown are non-significant (*p*-value > 0.05) and were analyzed in three biological replicates.

Regulation of 14 genes associated with secondary metabolism confirms the strong relevance of GPR8 for regulation of this process as indicated by the enrichment in this gene set as well. Among these genes are also the biosynthetic genes of the SOR-cluster *pks10s/sor2* and *pks11s/sor1*. Additionally, 21 transcription factor genes are down-regulated in Δ*gpr8* upon growth in darkness on cellulose. They include *ypr1* and *ypr2*, the transcription factors shown to have a major influence on the SOR cluster (Derntl et al., 2016; Monroy et al., 2017; Hitzenhammer et al., 2019). Concerning the SOR cluster, in addition to both PKS encoding genes and the transcription factor gene*s*, also the flavoprotein monooxygenase SOR5 and the isoamyl alcohol oxidase gene *sor7* are down-regulated in Δ*gpr8* on cellulose. Interestingly the deletion of *gpr8* does not significantly influence transcript levels of the SOR cluster in the presence of glucose in darkness (Figures 2A,B). Hence, the effect of GPR8 on the SOR cluster is likely to be mediated via YPR1 and/or YPR2 in a carbon source and light dependent manner.

Besides the SOR cluster (cluster 3), eight more clusters were found among the genes down-regulated in Δ*gpr8* on cellulose, of which four comprise genes associated with secondary metabolism, again strengthening the function of GPR8 in secondary metabolism.

### Genes Overlapping With YPR2 Targets

**FIGURE 3:**
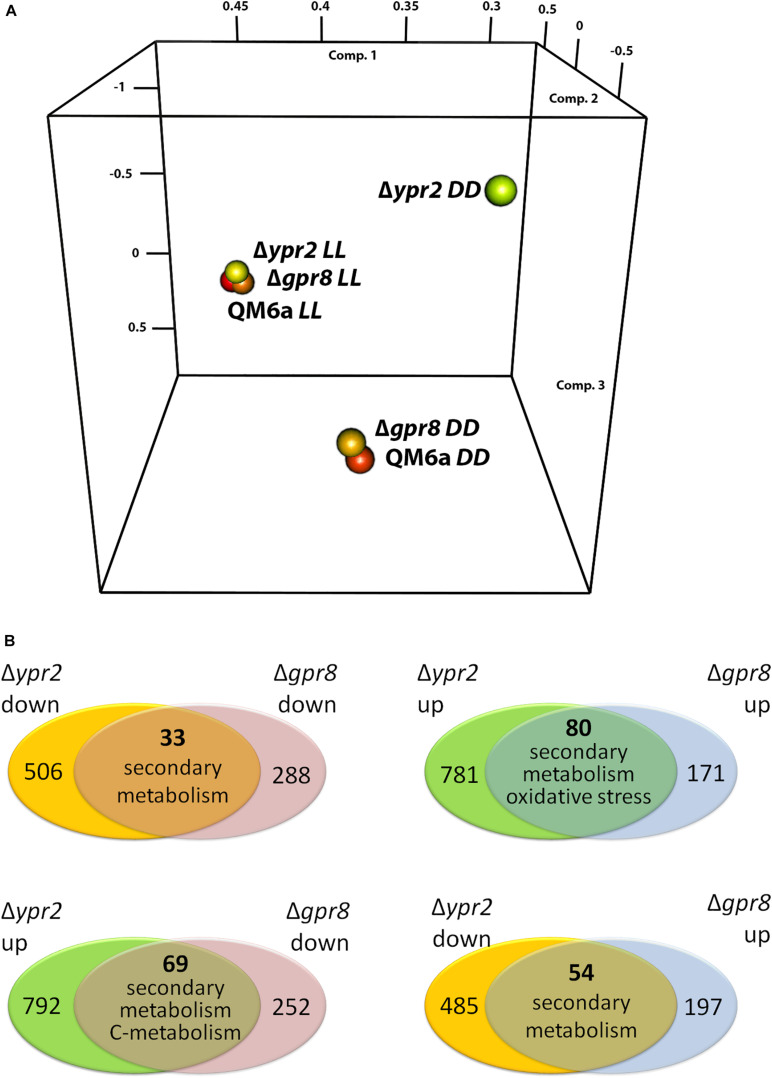
Regulatory relationships between strains lacking *gpr8* and those lacking *ypr2*. **(A)** Principal component analysis (PCA) of datasets for Δ*gpr8* and Δ*ypr2* upon growth in constant darkness (DD) or constant light (LL) on cellulose. **(B)** Consistent and contrasting regulatory overlaps between Δ*gpr8* and Δ*ypr2* upon growth in constant darkness on cellulose. Dominant functional categories of the overlapping genes are given for every comparison.

**FIGURE 4:**
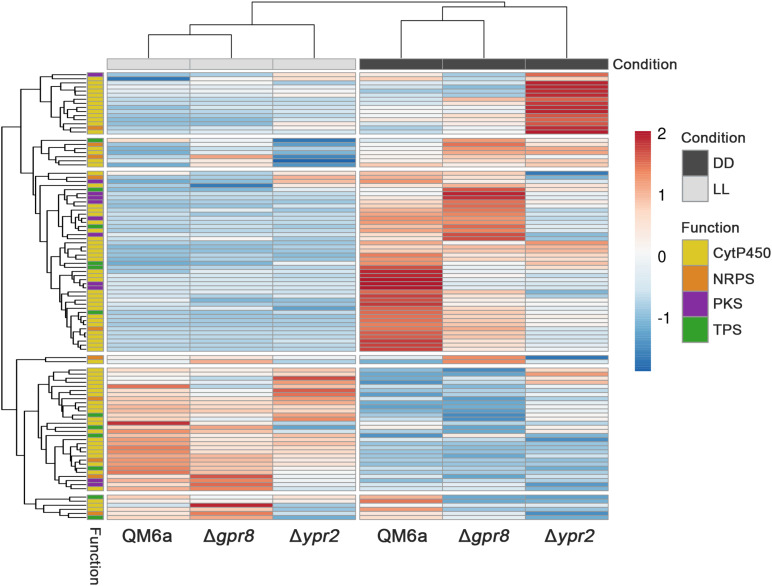
Heatmap of secondary metabolite synthase coding genes. CytP450 (cytochrome P450), NRPS (non-ribosomal peptide synthase), PKS (polyketide synthase) and TPS (terpene synthase) coding genes of wild type, Δ*gpr8* and Δ*ypr2* strains grown in light (LL) or darkness (DD) on cellulose are shown. A highresolution version of this figure comprising IDs of these genes is shown in Supplementary Material, Image 1.

Due to its genomic location in the vicinity of the SOR cluster, we were interested whether GPR8 shares regulatory targets with the transcription factor YPR2 upon growth on cellulose under controlled light conditions. Comparison of genes showing statistically significant regulation (*p*-value threshold 0.01; >2-fold regulation; (Hitzenhammer et al., 2019)) showed that in light none of the regulated genes overlaps between the targets of GPR8 and YPR2. Therefore we concentrated on darkness, where a PCA analysis showed a deviation from the wild type regulation pattern for both deletions (Figure 3A). Under these conditions we found that the overlap of regulated genes in the respective deletion strains was up to 47% of genes regulated in Δ*gpr8* (Figure 3B). Among the 33 genes down-regulated in Δ*gpr8* and Δ*ypr2* in darkness, functions in secondary metabolism were significantly enriched (*p*-value 3.3e-06). The same functions are enriched among the 80 genes up-regulated in Δ*gpr8* and Δ*ypr2* and additionally oxidative stress response (*p*-value 5.3e-07) and catalase reaction (*p*-value 1.09e-07). Interestingly, we also saw contrasting regulation between Δ*gpr8* and Δ*ypr2* with 69 genes up-regulated in Δ*ypr2* and down-regulated in Δ*gpr8*, which were again enriched in secondary metabolism (*p*-value 5.18e-04), but also in defense and virulence (*p*-value 7.54e-04) and in carbon metabolism (*p*-value 6.29e-04). The genes down-regulated in Δ*ypr2* and up-regulated in Δ*gpr8* showed enrichment in functions of secondary metabolism (*p*-value 2.14e-03) (Figure 3B and Supplementary Material, Table 3). Considering genes involved in biosynthesis of secondary metabolites, a clear influence of both *gpr8* and *ypr2* is observed (Figure 4 and Supplementary Material, Image 1). Besides an overlap in regulation, *ypr2* and *gpr8* both impact specific groups of synthases and clusters.

We conclude that the relevance of the signal transmitted by GPR8 for regulation by YPR2 is predominantly in adjustment of secondary metabolism, although the function of YPR2 is considerably broader under these conditions than that of GPR8.

### Regulation of Enzyme Production and Selected Secondary Metabolites by GPR8

The heterotrimeric G-protein pathway considerably impacts light modulated regulation of cellulase gene expression as well as post-transcriptional regulation of cellulases (Schmoll et al., 2016; Schmoll, 2018). Regulation of CAZyme gene expression by GPR8 is limited, but with an impact on transcript levels of for example *cel61b* or several candidate α and β-glycosidases, alterations in efficiency of cellulose degradation in Δ*gpr8* seemed possible. However, we detected no significant alterations in specific cellulase activity in Δ*gpr8* (data not shown).

**FIGURE 5:**
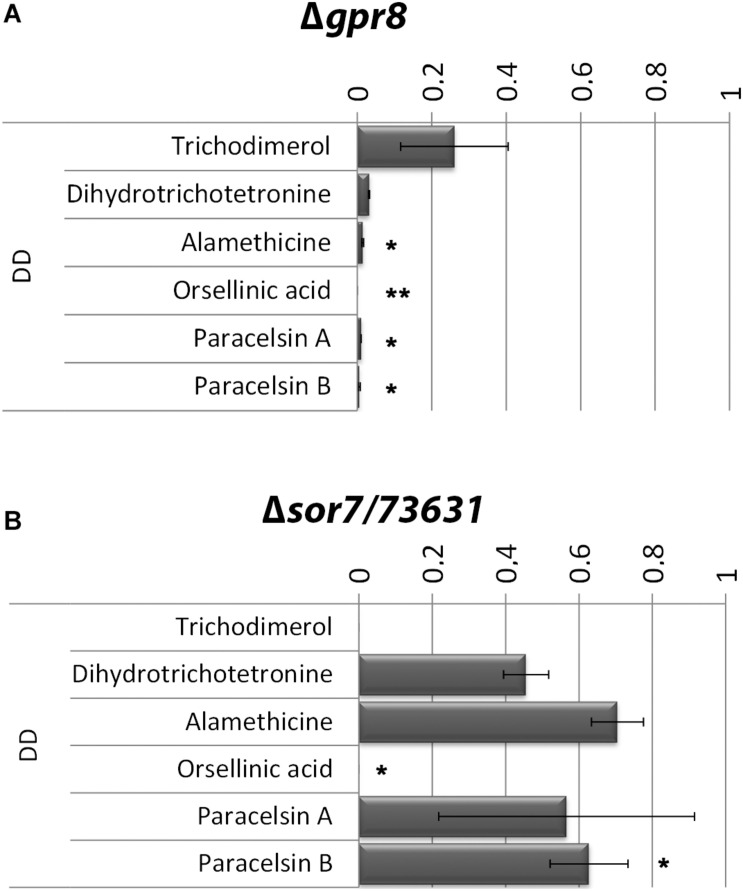
Secondary metabolite production in Δ*gpr8* and in Δ*sor7/TR_73631*. Mass spectrometric analysis and quantification with internal standards of culture supernatants of Δ*gpr8* **(A)** or Δ*sor7* **(B)** upon growth on cellulose in constant darkness (DD) relative to wild type. Values are normalized to produced biomass under these conditions. Error bars show standard deviations. Statistical significance was calculated using Student’s *T*-Test in R Studio (compare means, ggpubr v0.3.0). ** = *p*-value < 0.01; * = *p*-value < 0.05.

Based on the results of the transcriptome analysis we intended to evaluate the functions of GPR8 aimed at modulation of secondary metabolism in response to environmental signals. Therefore we analyzed supernatants from cellulose grown cultures of Δ*gpr8* for presence of selected metabolites using a mass spectrometric approach with internal standards. This analysis revealed a clear decrease in production of several secondary metabolites in Δ*gpr8* (Figure 5A). The decrease in abundance of trichodimerol and dihydrotrichotetronine is in accordance with the strongly decreased transcript levels of the SOR cluster genes in this mutant. On the other hand, a spectral analysis of the supernatant at 370 nm revealed no significant influence on the production of yellow sorbicillin derivatives in the presence of glucose. This supports a carbon source dependent function of GPR8 mediated signaling.

### Presence of *sor7*/TR_73631 Influences Production of Secondary Metabolites on Cellulose

The predicted FAD/FMN containing dehydrogenase SOR7 (Druzhinina et al., 2016a) was previously shown to be associated with the SOR cluster [(Derntl et al., 2017a) termed SOR4 in this study)] upon growth on glucose. Our analysis showed that *sor7* transcript levels are more than 20-fold lower in Δ*gpr8* compared to wild type in darkness on cellulose. Hence we analyzed whether the effect of GPR8 might be mediated in part by its impact on *sor7* levels. We deleted *sor7* in QM6a and validated the phenotype by crossing and growth assays of progeny carrying the mutation, which showed that the phenotype is propagated with the mutation (see also Materials and Methods). Deletion of *sor7* did not cause a growth defect in liquid culture in darkness or on plates. Growth was retarded on plates upon growth with carboxymethylcellulose or glucose as carbon source in light (Supplementary Material, Data Sheet 1, Figure S1). Indeed we found a decrease in Δ*sor7* for some compounds (Figure 5B), but the effect was less severe than that of GPR8. The dimeric sorbicillinoid trichodimerol is not produced in the Δ*sor7* mutant on cellulose, which is in agreement with the function of SOR7 in trichodimerol biosynthesis (Kahlert et al., 2020).

Transcript levels of the major cellulase gene *cbh1* are decreased to 41.3 ± 5.3% of wild type in Δ*sor7* on cellulose in darkness. Accordingly, specific cellulase activity is reduced to roughly the same extent (47.4 ± 5.0% of wild type) under the same conditions. These findings suggest that the impact of GPR8 may in part be mediated by alteration of SOR7 levels, but the relevance of SOR7 for cellulase formation indicates additional roles of this enzyme. Hence we consider this gene is not a major component of the signaling cascade targeted by GPR8.

## Discussion

**FIGURE 6:**
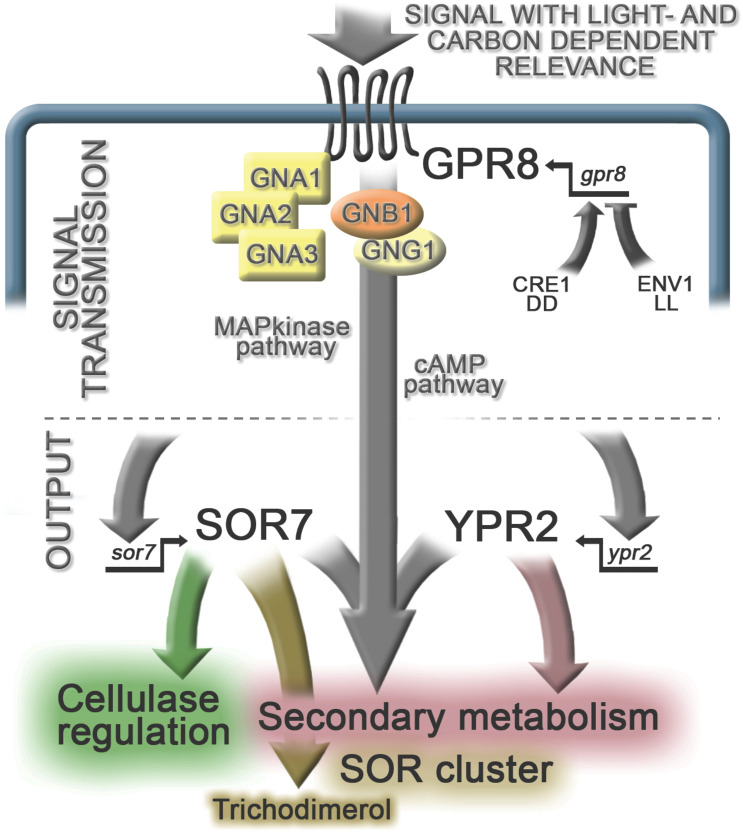
Schematic representation of the regulatory impact of GPR8. *Gpr8* transcript abundance is positively regulated by CRE1 in constant darkness (DD) and negatively by ENV1 in constant light (LL) upon growth on cellulose. Considering our results on gene regulation in the absence of GPR8 on glucose and cellulose in light and darkness, we conclude that the signal transmitted by GPR8 has a light- and carbon dependent relevance in *T. reesei*. Regulation by GPR8 is indirect and can be expected to involve the pathways for signal transmission depicted here as examples to be investigated. Signal transmission through such a cascade leads to the regulatory output: GPR8 positively impacts regulation of *ypr2*, but only partially shares its target genes upon growth on cellulose in darkness. *Sor7* transcript levels are positively influenced by GPR8 as well and SOR7 impacts production of several secondary metabolites, in addition to trichodimerol, for the biosynthesis of which it is essential. The presence of SOR7 also positively influences cellulase regulation through an as yet unknown mechanism contributing to the balance of secondary metabolism and enzyme production. The effect of GPR8 on secondary metabolism is assumed to be in part, but not exclusively mediated by its role in regulation of *ypr2* and *sor7*.

The genome of *T. reesei* comprises 57 genes encoding putative G-protein coupled receptors (Gruber et al., 2013; Schmoll et al., 2016). Of those only few have been characterized, confirming the functions of the predicted pheromone receptors (Seibel et al., 2012) and implicating the class XIII GPCRs CSG1 and CSG2 in glucose sensing (Stappler et al., 2017a). The heterotrimeric G-protein pathway with three G-alpha subunits, one G-beta and one G-gamma subunit was previously shown to impact cellulase gene expression, growth, cAMP signaling, conidiation and stress response (Schmoll et al., 2009; Seibel et al., 2009; Tisch et al., 2011a,b, 2014). Additionally, the role of the pheromone GPCRs HPR1 and HPR2 of *T. reesei* was shown for triggering sexual development in a mating type dependent manner (Seibel et al., 2012). However, as for most other fungi, the precise mechanism of the cascade operative to transmit the signal from these receptors to the output – for example transcription factors regulating cellulase gene expression – is not known yet. The cascade initiated by GPR8 first targets one or more of the three G-alpha subunits as well as the beta and gamma subunits. Downstream further signaling factors like MAP kinases or the cAMP pathway contribute to the output and on the way, numerous factors modulate signal transmission (Figure 6). In the model organism *Saccharomyces cerevisiae*, one of the rare cases of detailed investigation of G-protein signaling out put was achieved over decades of research, which impressively shows the complexity of signaling, with different contributing pathways as well as feedback mechanisms operative (Alvaro and Thorner, 2016). Considering this complexity, we see our study as a first step in investigation of external signals and their reception for modulation of secondary metabolism. Clearly, the role of GPR8 in regulation of secondary metabolism will be an indirect one and as our results show, it likely involves influence on multiple secondary metabolite clusters and their cognate transcription factors.

A role in secondary metabolism was not previously studied for the G-protein pathway in *T. reesei*. However, the genomic locus of *gpr8* close to the SOR cluster as well as its regulation by CRE1 in a light dependent manner (Monroy et al., 2017) prompted us to study its function. In accordance with the earlier studies of G-protein signaling, also the function of GPR8 was specific to a light condition – in this case darkness. Additionally, G-protein alpha subunits were previously only known for the light dependent function in cellulase gene expression, which applies to all three subunits (Schmoll et al., 2009; Seibel et al., 2009; Rodriguez-Iglesias, unpublished) with the function of GPR8 in secondary metabolism, but not cellulase gene expression, a functional specificity of heterotrimeric G-protein signaling depending on the signal becomes obvious, which not necessarily targets enzyme regulation. We conclude that the signal received by GPR8 has a light dependent relevance for *T. reesei*, influencing secondary metabolism in darkness rather than in light.

Comparison of direct or indirect gene regulation by YPR2 (Hitzenhammer et al., 2019) with that influenced by the cascade initiated by GPR8 showed sizable overlaps. Interestingly, overlapping regulation was only found in darkness and was both consistent (up or downregulation in mutant strains) or contrasting (up-regulation in one, downregulation in the other mutant strain). Consequently, it can be assumed that the signal transmitted by GPR8 initiates a cascade that involves the function of YPR2. This assumption is supported by the fact that *ypr2* is down-regulated in Δ*gpr8* in darkness (Supplementary Material, Table 2). However, also the clear differences in the regulation patterns between regulatory targets of YPR2 and GPR8 (Figure 2A) reflect that both factors have distinct functions in regulation of secondary metabolism, which only partially overlap. Additionally, while GPR8 and YPR2 do show a considerable number of targets with functions in secondary metabolism, our data indicate that the signaling cascade of GPR8 is not exclusively targeted at YPR2, but has additional and divergent targets as well. The effect of GPR8 on *sor7*/TR_73631 is not detected upon lack of YPR2, in which case transcript levels of *sor7* show only a minor alteration compared to wild type (Hitzenhammer et al., 2019), hence representing such a divergent case. Interestingly, *ypr1*, the second crucial transcription factor in the SOR cluster is more than 100-fold more abundant upon growth on glucose than on cellulose (our own unpublished data) and positively regulated by YPR2 and GPR8 on cellulose, albeit on a very low level. Also since we do not see a regulatory function of GPR8 on the SOR cluster on glucose, we assume that in the cascade initiated after signal reception by GPR8, YPR1 does not play a major role.

Recently, the homolog of *T. reesei* GPR8 in *Aspergillus fumigatus*, GprM, was found to be a negative regulator of sterigmatocystin biosynthesis (Dos Reis et al., 2019), which, however, does not allow for a general estimation of the influence of GprM on secondary metabolism. In contrast, our data show a positive effect of GPR8 on gene regulation in secondary metabolism on cellulose. Although also a divergent function of the receptors in *A. fumigatus* and *T. reesei* is possible, the use of different carbon sources in the two experiments may be a reason as well. While for *T. reesei* most effects were seen on cellulose as carbon source, the cultivations for *A. fumigatus* were likely done with another carbon source, likely glucose, for which we observed no regulation of the SOR cluster. Differences in regulation of secondary metabolism between growth on glucose and cellulose, particularly with respect to the SOR cluster became obvious previously also in *T. reesei* (Derntl et al., 2016; Monroy et al., 2017). Here we also found a clear regulatory impact on the SOR cluster genes on cellulose, which was not detectable on glucose, hence confirming a different relevance of the signal received by GPR8 upon growth on cellulose versus glucose.

DHN melanin production was reported to be negatively influenced by GprM in *A. fumigatus* (Manfiolli et al., 2019). Melanin production is not well studied in *Trichoderma* spp. Melanin is reported to be a constituent of the spore wall of *Trichoderma viride* (Benitez et al., 1976), thereby potentially protecting the spore from lysis (Bull, 1970). However, of the genes reported to be responsible for melanin production in *A. fumigatus* (Perez-Cuesta et al., 2020), only *pksP* and *abr2* were found to have direct homologs in *T. reesei* (TR_82208/*pks4* and TR_124079). Both genes are up-regulated in Δ*gpr8* in darkness in *T. reesei*. *Pks4* is responsible for the green pigmentation of spores of *T. reesei* and its deletion led to an alteration of expression levels of other polyketide synthase genes (Atanasova et al., 2013). In our study we did not observe a visible alteration in spore pigmentation in the deletion strain of *gpr8*. Regulation of two PKS encoding genes was found in Δ*gpr8* which diverges from the result of deletion of *pks4*. Hence it is unlikely that the effects seen in secondary metabolism caused by deletion of *gpr8* are due to an altered regulation of *pks4*.

With the influence of GPR8 on abundance of *sor7*, which is relevant for cellulase regulation, we see a further example of an interrelationship between regulation of secondary metabolism. A function in balancing primary and secondary metabolism was postulated for the transcription factor XPP1 (Derntl et al., 2017b), however, its encoding gene is not regulated by YPR2 or GPR8. Further interesting factors for which roles in secondary metabolism and cellulase regulation were reported include VEL1 (Karimi Aghcheh et al., 2014) and LAE1 (Seiboth et al., 2012) and some protein phosphatases (Rodriguez-Iglesias and Schmoll, 2019), none of which are targets of GPR8. While indications for a regulated balance between secondary metabolism and nutrient acquisition keep accumulating, it still remains to be discovered how this interaction is mediated at the mechanistic level.

## Conclusion

In summary, our study revealed the first G-protein coupled receptor in *T. reesei*, that impacts secondary metabolism (Figure 6). We found that its function targets not only the SOR cluster, but influences secondary metabolism more broadly, likely targeting multiple regulators. Besides YPR2, one transcription factor of the SOR cluster, GPR8 further impacts a regulator of sor7/TR_73631, which is not regulated by YPR2. Thereby we provide insight into the complex regulation of secondary metabolism by environmental signals in *T. reesei*.

## Data Availability Statement

The datasets presented in this study are included in this article and its additional files. GenBank Accession numbers for datasets analyzed for this study are given in Methods and described in the respective cited articles.

## Author Contributions

WH performed RT-qPCR, phenotyping of fertile derivatives, statistical analyses and contributed to analysis of gene expression, and drafting of the manuscript. SB contributed to analysis of gene expression and RT-qPCR. AM contributed to analysis of cellulase regulation and RNA isolation, HB contributed to bioinformatic analysis. CD performed the gene deletion. MS conceived of the study, contributed to bioinformatic analysis, interpretation of results and wrote the final version of the manuscript. All authors contributed to the article and approved the submitted version.

## Conflict of Interest

HB was employed by the company Symbiocyte. The remaining authors declare that the research was conducted in the absence of any commercial or financial relationships that could be construed as a potential conflict of interest.

## Footnotes

1 https://genome.jgi.doe.gov/Trire2/Trire2.home.html

2 https://sbi.hki-jena.de/fungifun/

3 https://mycocosm.jgi.doe.gov/Trire2/Trire2.home.html

## References

[B1] Adnan M. Zheng W. Islam W. Arif M. Abubakar Y. S. Wang Z. (2017). Carbon catabolite repression in filamentous fungi. *Int. J. Mol. Sci.* 19:48. 10.3390/ijms19010048 29295552 PMC5795998

[B2] Alvaro C. G. Thorner J. (2016). Heterotrimeric G Protein-coupled Receptor signaling in yeast mating pheromone response. *J. Biol. Chem.* 291 7788–7795. 10.1074/jbc.R116.714980 26907689 PMC4824985

[B3] Atanasova L. Knox B. P. Kubicek C. P. Druzhinina I. S. Baker S. E. (2013). The polyketide synthase gene pks4 of *Trichoderma reesei* provides pigmentation and stress resistance. *Eukaryot. Cell.* 12 1499–1508. 10.1128/EC.00103-13 24036343 PMC3837940

[B4] Bazafkan H. Beier S. Stappler E. Böhmdorfer S. Oberlerchner J. T. Sulyok M. (2017a). SUB1 has photoreceptor dependent and independent functions in sexual development and secondary metabolism in *Trichoderma reesei*. *Mol. Microbiol.* 106 742–759. 10.1111/mmi.13842 28925526

[B5] Bazafkan H. Dattenbock C. Stappler E. Beier S. Schmoll M. (2017b). Interrelationships of VEL1 and ENV1 in light response and development in *Trichoderma reesei*. *PLoS One* 12:e0175946. 10.1371/journal.pone.0175946 28423024 PMC5397039

[B6] Bazafkan H. Dattenböck C. Böhmdorfer S. Tisch D. Stappler E. Schmoll M. (2015). Mating type dependent partner sensing as mediated by VEL1 in *Trichoderma reesei*. *Mol. Microbiol.* 96 1103–1118. 10.1111/mmi.12993 25757597 PMC4949666

[B7] Bazafkan H. Tisch D. Schmoll M. (2014). “Regulation of glycoside hydrolase expression in *Trichoderma*,” in *Biotechnology and Biology of Trichoderma*, eds Gupta V. K. Schmoll M. Herrera-Estrella A. Upadhyay R. S. Druzhinina I. Tuohy M. G. (Oxford, UK: Elsevier), 291–307. 10.1016/b978-0-444-59576-8.00020-5

[B8] Benitez T. Villa T. G. Garcia Acha I. (1976). Some chemical and structural features of the conidial wall of *Trichoderma viride*. *Can. J. Microbiol.* 22 318–321. 10.1139/m76-046 944079

[B9] Benocci T. Aguilar-Pontes M. V. Zhou M. Seiboth B. de Vries R. P. (2017). Regulators of plant biomass degradation in ascomycetous fungi. *Biotechnol. Biofuels* 10:152. 10.1186/s13068-017-0841-x 28616076 PMC5468973

[B10] Bull A. T. (1970). Inhibition of polysaccharases by melanin: enzyme inhibition in relation to mycolysis. *Arch. Biochem. Biophys.* 137 345–356. 10.1016/0003-9861(70)90448-0 4191418

[B11] Cabrera I. E. Pacentine I. V. Lim A. Guerrero N. Krystofova S. Li L. (2015). Global analysis of predicted G protein-coupled receptor genes in the filamentous fungus, *Neurospora crassa*. *G3* 5 2729–2743. 10.1534/g3.115.020974 26464358 PMC4683645

[B12] Dattenböck C. Tisch D. Schuster A. Monroy A. A. Hinterdobler W. Schmoll M. (2018). Gene regulation associated with sexual development and female fertility in different isolates of *Trichoderma reesei*. *Fungal Biol. Biotechnol.* 5:9. 10.1186/s40694-018-0055-4 PMC5952832 29785273

[B13] Dekhang R. Wu C. Smith K. M. Lamb T. M. Peterson M. Bredeweg E. L. (2017). The *Neurospora* transcription factor ADV-1 transduces light signals and temporal information to control rhythmic expression of genes involved in cell fusion. *G3* 7 129–142. 10.1534/g3.116.034298 27856696 PMC5217103

[B14] Derntl C. Guzman-Chavez F. Mello-de-Sousa T. M. Busse H. J. Driessen A. J. M. Mach R. L. (2017a). In vivo study of the sorbicillinoid gene cluster in *Trichoderma reesei*. *Front. Microbiol.* 8:2037. 10.3389/fmicb.2017.02037 29104566 PMC5654950

[B15] Derntl C. Kluger B. Bueschl C. Schuhmacher R. Mach R. L. Mach-Aigner A. R. (2017b). Transcription factor Xpp1 is a switch between primary and secondary fungal metabolism. *Proc. Natl. Acad. Sci. U.S.A.* 114 E560–E569. 10.1073/pnas.1609348114 28074041 PMC5278490

[B16] Derntl C. Rassinger A. Srebotnik E. Mach R. L. Mach-Aigner A. R. (2016). Identification of the main regulator responsible for synthesis of the typical yellow pigment produced by *Trichoderma reesei*. *Appl. Environ. Microbiol.* 82 6247–6257. 10.1128/AEM.01408-16 27520818 PMC5068150

[B17] Dos Reis T. F. Mellado L. Lohmar J. M. Silva L. P. Zhou J. J. Calvo A. M. (2019). GPCR-mediated glucose sensing system regulates light-dependent fungal development and mycotoxin production. *PLoS Genet.* 15:e1008419. 10.1371/journal.pgen.1008419 31609971 PMC6812930

[B18] Druzhinina I. S. Kopchinskiy A. G. Kubicek E. M. Kubicek C. P. (2016a). A complete annotation of the chromosomes of the cellulase producer *Trichoderma reesei* provides insights in gene clusters, their expression and reveals genes required for fitness. *Biotechnol. Biofuels* 9:75. 10.1186/s13068-016-0488-z 27030800 PMC4812632

[B19] Druzhinina I. S. Kubicek E. M. Kubicek C. P. (2016b). Several steps of lateral gene transfer followed by events of ‘birth-and-death’ evolution shaped a fungal sorbicillinoid biosynthetic gene cluster. *BMC Evol. Biol.* 16:269. 10.1186/s12862-016-0834-6 28010735 PMC5182515

[B20] Frisvad J. C. Moller L. L. H. Larsen T. O. Kumar R. Arnau J. (2018). Safety of the fungal workhorses of industrial biotechnology: update on the mycotoxin and secondary metabolite potential of *Aspergillus niger*, *Aspergillus oryzae*, and *Trichoderma reesei*. *Appl. Microbiol. Biotechnol.* 102 9481–9515. 10.1007/s00253-018-9354-1 30293194 PMC6208954

[B21] Glass N. L. Schmoll M. Cate J. H. Coradetti S. (2013). Plant cell wall deconstruction by ascomycete fungi. *Annu. Rev. Microbiol.* 67 477–498. 10.1146/annurev-micro-092611-150044 23808333

[B22] Gruber F. Visser J. Kubicek C. P. de Graaff L. H. (1990). The development of a heterologous transformation system for the cellulolytic fungus *Trichoderma reesei* based on a pyrG-negative mutant strain. *Curr. Genet.* 18 71–76. 10.1007/bf00321118 2245476

[B23] Gruber S. Omann M. Zeilinger S. (2013). Comparative analysis of the repertoire of G protein-coupled receptors of three species of the fungal genus *Trichoderma*. *BMC Microbiol.* 13:108. 10.1186/1471-2180-13-108 23679152 PMC3664084

[B24] Guzman-Chavez F. Salo O. Nygard Y. Lankhorst P. P. Bovenberg R. A. L. Driessen A. J. M. (2017). Mechanism and regulation of sorbicillin biosynthesis by *Penicillium chrysogenum*. *Microb. Biotechnol.* 10 958–968. 10.1111/1751-7915.12736 28618182 PMC5481523

[B25] Hitzenhammer E. Büschl C. Sulyok M. Schuhmacher R. Kluger B. Wischnitzki E. (2019). YPR2 is a regulator of light modulated carbon and secondary metabolism in *Trichoderma reesei*. *BMC Genomics* 20:211. 10.1186/s12864-019-5574-8 30866811 PMC6417087

[B26] Kahlert L. Bassiony E. F. Cox R. J. Skellam E. J. (2020). Diels-alder reactions during the biosynthesis of sorbicillinoids. *Angew. Chem. Int. Ed. Engl.* 59 5816–5822. 10.1002/anie.201915486 31943627 PMC7154774

[B27] Karimi Aghcheh R. Nemeth Z. Atanasova L. Fekete E. Paholcsek M. Sandor E. (2014). The VELVET A orthologue VEL1 of *Trichoderma reesei* regulates fungal development and is essential for cellulase gene expression. *PLoS One* 9:e112799. 10.1371/journal.pone.0112799 25386652 PMC4227869

[B28] Kiesenhofer D. Mach-Aigner A. R. Mach R. L. (2016). “Understanding the mechanism of carbon catabolite repression to increase protein production in filamentous fungi,” in *Gene Expression Systems in Fungi: Advancements and Applications*, eds Schmoll M. Dattenböck D. (Switzerland: Springer International Publishing), 275–288. 10.1007/978-3-319-27951-0_12

[B29] Lafon A. Han K. H. Seo J. A. Yu J. H. d’Enfert C. (2006). G-protein and cAMP-mediated signaling in *Aspergilli*: a genomic perspective. *Fungal Genet. Biol.* 43 490–502. 10.1016/j.fgb.2006.02.001 16546420

[B30] Li C. Lin F. Sun W. Yuan S. Zhou Z. Wu F. G. (2018). Constitutive hyperproduction of sorbicillinoids in *Trichoderma reesei* ZC121. *Biotechnol. Biofuels* 11:291. 10.1186/s13068-018-1296-4 30386428 PMC6202828

[B31] Li H. Durbin R. (2009). Fast and accurate short read alignment with Burrows-Wheeler transform. *Bioinformatics* 25 1754–1760. 10.1093/bioinformatics/btp324 19451168 PMC2705234

[B32] Li H. Handsaker B. Wysoker A. Fennell T. Ruan J. Homer N. (2009). The Sequence Alignment/Map format and SAMtools. *Bioinformatics* 25 2078–2079. 10.1093/bioinformatics/btp352 19505943 PMC2723002

[B33] Li L. Wright S. J. Krystofova S. Park G. Borkovich K. A. (2007). Heterotrimeric G protein signaling in filamentous fungi. *Annu. Rev. Microbiol.* 61 423–452. 10.1146/annurev.micro.61.080706.093432 17506673

[B34] Li W. C. Huang C. H. Chen C. L. Chuang Y. C. Tung S. Y. Wang T. F. (2017). *Trichoderma reesei* complete genome sequence, repeat-induced point mutation, and partitioning of CAZyme gene clusters. *Biotechnol. Biofuels* 10:170. 10.1186/s13068-017-0825-x 28690679 PMC5496416

[B35] Linke R. Thallinger G. G. Haarmann T. Eidner J. Schreiter M. Lorenz P. (2015). Restoration of female fertility in *Trichoderma reesei* QM6a provides the basis for inbreeding in this industrial cellulase producing fungus. *Biotechnol. Biofuels* 8:155. 10.1186/s13068-015-0311-2 26405457 PMC4581161

[B36] Liu D. Coloe S. Baird R. Pederson J. (2000). Rapid mini-preparation of fungal DNA for PCR. *J. Clin. Microbiol.* 38:471. 10.1128/jcm.38.1.471-471.2000 PMC88759 10681211

[B37] Malachova A. Sulyok M. Beltran E. Berthiller F. Krska R. (2014). Optimization and validation of a quantitative liquid chromatography-tandem mass spectrometric method covering 295 bacterial and fungal metabolites including all regulated mycotoxins in four model food matrices. *J. Chromatogr. A* 1362 145–156. 10.1016/j.chroma.2014.08.037 25175039

[B38] Mandels M. Andreotti R. (1978). Problems and challenges in the cellulose to cellulase fermentation. *Proc. Biochem.* 13 6–13.

[B39] Manfiolli A. O. Siqueira F. S. Dos Reis T. F. Van Dijck P. Schrevens S. Hoefgen S. (2019). Mitogen-activated protein kinase cross-talk interaction modulates the mroduction of melanins in *Aspergillus fumigatus*. *mBio* 10:e00215-19. 10.1128/mBio.00215-19 30914505 PMC6437049

[B40] Marie-Nelly H. Marbouty M. Cournac A. Flot J. F. Liti G. Parodi D. P. (2014). High-quality genome (re)assembly using chromosomal contact data. *Nat. Commun.* 5:5695. 10.1038/ncomms6695 25517223 PMC4284522

[B41] Martinez D. Berka R. M. Henrissat B. Saloheimo M. Arvas M. Baker S. E. (2008). Genome sequencing and analysis of the biomass-degrading fungus *Trichoderma reesei* (syn. *Hypocrea jecorina*). *Nat. Biotechnol.* 26 553–560. 18454138 10.1038/nbt1403

[B42] Metsalu T. Vilo J. (2015). ClustVis: a web tool for visualizing clustering of multivariate data using principal component analysis and heatmap. *Nucleic Acids Res.* 43 W566–W570. 10.1093/nar/gkv468 25969447 PMC4489295

[B43] Monroy A. A. Stappler E. Schuster A. Sulyok M. Schmoll M. (2017). A CRE1- regulated cluster is responsible for light dependent production of dihydrotrichotetronin in *Trichoderma reesei*. *PLoS One* 12:e0182530. 10.1371/journal.pone.0182530 28809958 PMC5557485

[B44] Nevalainen H. Suominen P. Taimisto K. (1994). On the safety of *Trichoderma reesei*. *J. Biotechnol.* 37 193–200. 10.1016/0168-1656(94)90126-0 7765573

[B45] Perez-Cuesta U. Aparicio-Fernandez L. Guruceaga X. Martin-Souto L. Abad-Diaz-de-Cerio A. Antoran A. (2020). Melanin and pyomelanin in *Aspergillus fumigatus*: from its genetics to host interaction. *Int. Microbiol.* 23 55–63. 10.1007/s10123-019-00078-0 31020477

[B46] Priebe S. Kreisel C. Horn F. Guthke R. Linde J. (2015). FungiFun2: a comprehensive online resource for systematic analysis of gene lists from fungal species. *Bioinformatics* 31 445–446. 10.1093/bioinformatics/btu627 25294921 PMC4308660

[B47] Ritchie M. E. Phipson B. Wu D. Hu Y. Law C. W. Shi W. (2015). limma powers differential expression analyses for RNA-sequencing and microarray studies. *Nucleic Acids Res.* 43:e47. 10.1093/nar/gkv007 25605792 PMC4402510

[B48] Rodriguez-Iglesias A. Schmoll M. (2019). Protein phosphatases regulate growth, development, cellulases and secondary metabolism in *Trichoderma reesei*. *Sci. Rep.* 9:10995. 10.1038/s41598-019-47421-z 31358805 PMC6662751

[B49] Salo O. Guzman-Chavez F. Ries M. I. Lankhorst P. P. Bovenberg R. A. Vreeken R. J. (2016). Identification of a polyketide synthase involved in sorbicillin biosynthesis by *Penicillium chrysogenum*. *Appl. Environ. Microbiol.* 82 3971–3978. 10.1128/AEM.00350-16 27107123 PMC4907180

[B50] Schmoll M. (2013). “Sexual development in *Trichoderma* - scrutinizing the aspired phenomenon,” in *Trichoderma: Biology and Applications*, eds Mukherjee P. K. Horwitz B. A. Singh U. S. Mukherjee M. Schmoll M. (London: CAB International), 67–86. 10.1079/9781780642475.0067

[B51] Schmoll M. (2018). Regulation of plant cell wall degradation by light in *Trichoderma*. *Fungal Biol. Biotechnol.* 5:10. 10.1186/s40694-018-0052-7 29713489 PMC5913809

[B52] Schmoll M. Dattenböck C. Carreras-Villasenor N. Mendoza-Mendoza A. Tisch D. Aleman M. I. (2016). The genomes of three uneven siblings: footprints of the lifestyles of three *Trichoderma* species. *Microbiol. Mol. Biol. Rev.* 80 205–327. 10.1128/MMBR.00040-15 26864432 PMC4771370

[B53] Schmoll M. Franchi L. Kubicek C. P. (2005). Envoy, a PAS/LOV domain protein of *Hypocrea jecorina* (Anamorph *Trichoderma reesei*), modulates cellulase gene transcription in response to light. *Eukaryot Cell* 4 1998–2007. 10.1128/ec.4.12.1998-2007.2005 16339718 PMC1317494

[B54] Schmoll M. Schuster A. do Nascimento Silva R. Kubicek C. P. (2009). The G-alpha protein GNA3 of *Hypocrea jecorina* (anamorph *Trichoderma reesei*) regulates cellulase gene expression in the presence of light. *Eukaryot Cell* 8 410–420. 10.1128/EC.00256-08 19136572 PMC2653238

[B55] Schmoll M. Zeilinger S. Mach R. L. Kubicek C. P. (2004). Cloning of genes expressed early during cellulase induction in *Hypocrea jecorina* by a rapid subtraction hybridization approach. *Fungal Genet. Biol.* 41 877–887. 10.1016/j.fgb.2004.06.002 15288024

[B56] Schuster A. Bruno K. S. Collett J. R. Baker S. E. Seiboth B. Kubicek C. P. (2012). A versatile toolkit for high throughput functional genomics with *Trichoderma reesei*. *Biotechnol. Biofuels* 5:1. 10.1186/1754-6834-5-1 22212435 PMC3260098

[B57] Seibel C. Gremel G. Silva R. D. Schuster A. Kubicek C. P. Schmoll M. (2009). Light-dependent roles of the G-protein subunit GNA1 of *Hypocrea jecorina* (anamorph *Trichoderma reesei*). *BMC Biol.* 7:58. 10.1186/1741-7007-7-58 19728862 PMC2749820

[B58] Seibel C. Tisch D. Kubicek C. P. Schmoll M. (2012). The role of pheromone receptors for communication and mating in *Hypocrea jecorina* (*Trichoderma reesei*). *Fungal Genet. Biol.* 49 814–824. 10.1016/j.fgb.2012.07.004 22884620 PMC3462998

[B59] Seiboth B. Karimi R. A. Phatale P. A. Linke R. Hartl L. Sauer D. G. (2012). The putative protein methyltransferase LAE1 controls cellulase gene expression in *Trichoderma reesei*. *Mol. Microbiol.* 84 1150–1164. 10.1111/j.1365-2958.2012.08083.x 22554051 PMC3370264

[B60] Seidl V. Seibel C. Kubicek C. P. Schmoll M. (2009). Sexual development in the industrial workhorse *Trichoderma reesei*. *Proc. Natl. Acad. Sci. U.S.A.* 106 13909–13914. 10.1073/pnas.0904936106 19667182 PMC2728994

[B61] Seo J. Gordish-Dressman H. Hoffman E. P. (2006). An interactive power analysis tool for microarray hypothesis testing and generation. *Bioinformatics* 22 808–814. 10.1093/bioinformatics/btk052 16418236

[B62] Stappler E. Dattenböck C. Tisch D. Schmoll M. (2017a). Analysis of light- and carbon-specific transcriptomes implicates a class of G-protein-coupled receptors in cellulose sensing. *mSphere* 2:e00089-17. 10.1128/mSphere.00089-17 28497120 PMC5425790

[B63] Stappler E. Walton J. D. Schmoll M. (2017b). Abundance of secreted proteins of *Trichoderma reesei* is regulated by light of different intensities. *Front. Microbiol.* 8:2586. 10.3389/fmicb.2017.02586 29375497 PMC5770571

[B64] Sulyok M. Krska R. Schuhmacher R. (2007). A liquid chromatography/tandem mass spectrometric multi-mycotoxin method for the quantification of 87 analytes and its application to semi-quantitative screening of moldy food samples. *Anal. Bioanal. Chem.* 389 1505–1523. 10.1007/s00216-007-1542-2 17874237

[B65] Tian C. Beeson W. T. Iavarone A. T. Sun J. Marletta M. A. Cate J. H. (2009). Systems analysis of plant cell wall degradation by the model filamentous fungus *Neurospora crassa*. *Proc. Natl. Acad. Sci. U.S.A.* 106 22157–22162. 10.1073/pnas.0906810106 20018766 PMC2794032

[B66] Tisch D. Kubicek C. P. Schmoll M. (2011a). New insights into the mechanism of light modulated signaling by heterotrimeric G-proteins: ENVOY acts on gna1 and gna3 and adjusts cAMP levels in *Trichoderma reesei* (*Hypocrea jecorina*). *Fungal Genet. Biol.* 48 631–640. 10.1016/j.fgb.2010.12.009 21220037 PMC3082050

[B67] Tisch D. Kubicek C. P. Schmoll M. (2011b). The phosducin-like protein PhLP1 impacts regulation of glycoside hydrolases and light response in *Trichoderma reesei*. *BMC Genomics* 12:613. 10.1186/1471-2164-12-613 PMC3267782 22182583

[B68] Tisch D. Pomraning K. R. Collett J. R. Freitag M. Baker S. E. Chen C. L. (2017). Omics analyses of *Trichoderma reesei* CBS999.97 and QM6a indicate the relevance of female fertility to carbohydrate-active enzyme and transporter levels. *Appl. Environ. Microbiol.* 83:e01578-17. 10.1128/AEM.01578-17 28916559 PMC5666144

[B69] Tisch D. Schmoll M. (2013). Targets of light signalling in *Trichoderma reesei*. *BMC Genomics* 14:657. 10.1186/1471-2164-14-657 24070552 PMC3831817

[B70] Tisch D. Schuster A. Schmoll M. (2014). Crossroads between light response and nutrient signalling: ENV1 and PhLP1 act as mutual regulatory pair in *Trichoderma reesei*. *BMC Genomics* 15:425. 10.1186/1471-2164-15-425 24893562 PMC4076981

