# Supplementary material for: The G-protein Coupled Receptor GPR8 Regulates Secondary Metabolism in Trichoderma reesei
Source: Front Bioeng Biotechnol. 2020 Nov 5;8:558996. doi: 10.3389/fbioe.2020.558996 (PMC7676458; doi:10.3389/fbioe.2020.558996)
Supplement: Supplementary file 1 [file Data_Sheet_1.PDF]

The G-protein coupled receptor GPR8 regulates secondary metabolism in *Trichoderma reesei*

Wolfgang Hinterdobler[1], Sabrina Beier[1], Alberto Alonso Monroy[1], Harald Berger[2], Christoph Dattenböck[1] and Schmoll Monika[1]*

Supplementary material

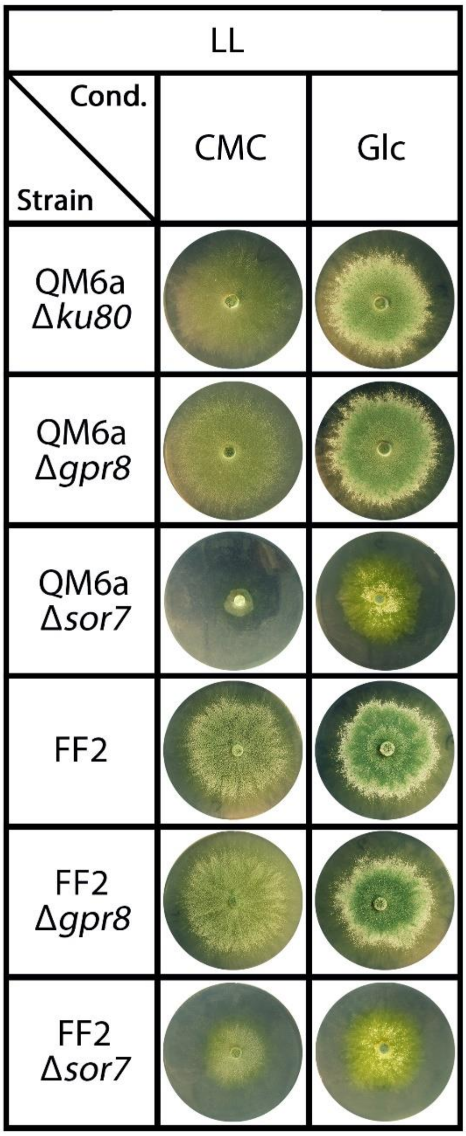

Figure S1. **Phenotypes of Δ*gpr8* and Δ*sor7* upon growth on cellulose and glucose.** Strains lacking *gpr8* or *sor7* in the background of QM6a were crossed with female fertile MAT1-2 strains with largely similar background (Bazafkan et al., 2015 Mol Microbiol 96(6):1103; Tisch et al., 2017 Appl Environ Microbiol 83(22):e01578-17). Progeny lacking the respective genes and having the same mating type as QM6a (MAT1-2, FF2 in the figure) were analyzed for altered phenotypes upon growth on Mandels Andreotti minimal medium with 1 % (w/v) carboxymethyl cellulose (CMC) and glucose (Glc) for 72 hours at 1700 lux (LL). The growth defect of the sor7 deletion strain observed in QM6a background is propagated with the mutation after crossing.

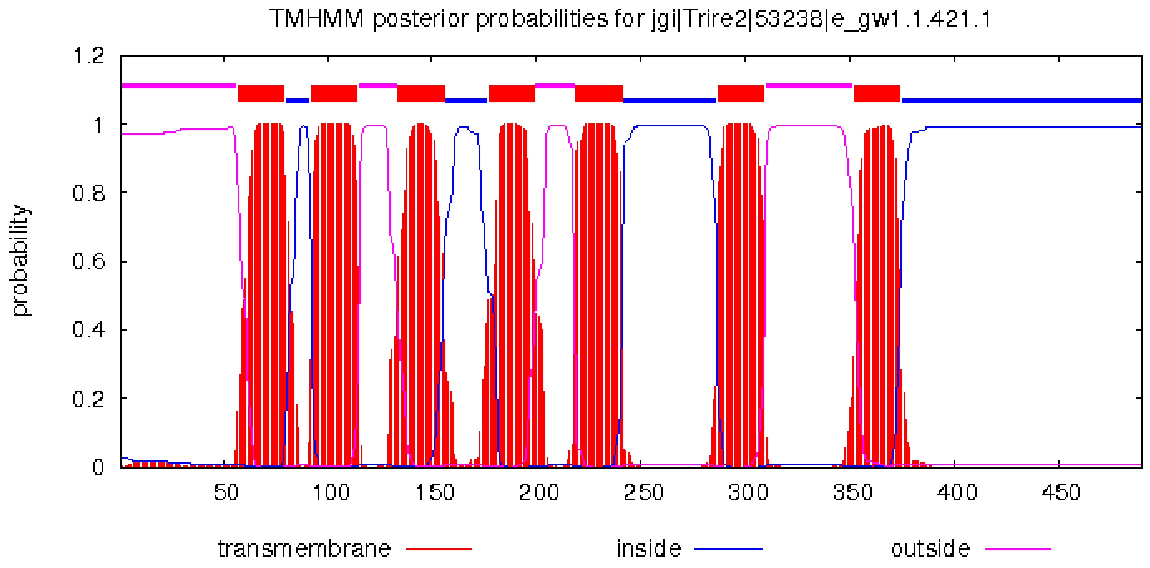

Figure S2. **TMHMM analysis of GPR8.** The online tool TMHMM available at DTU (online server for analysis: http://www.cbs.dtu.dk/services/TMHMM/) was used for this analysis. Transmembrane domain analysis revealed intracellular (blue) extracellular (pink) and transmembrane (red) areas of GPR8.

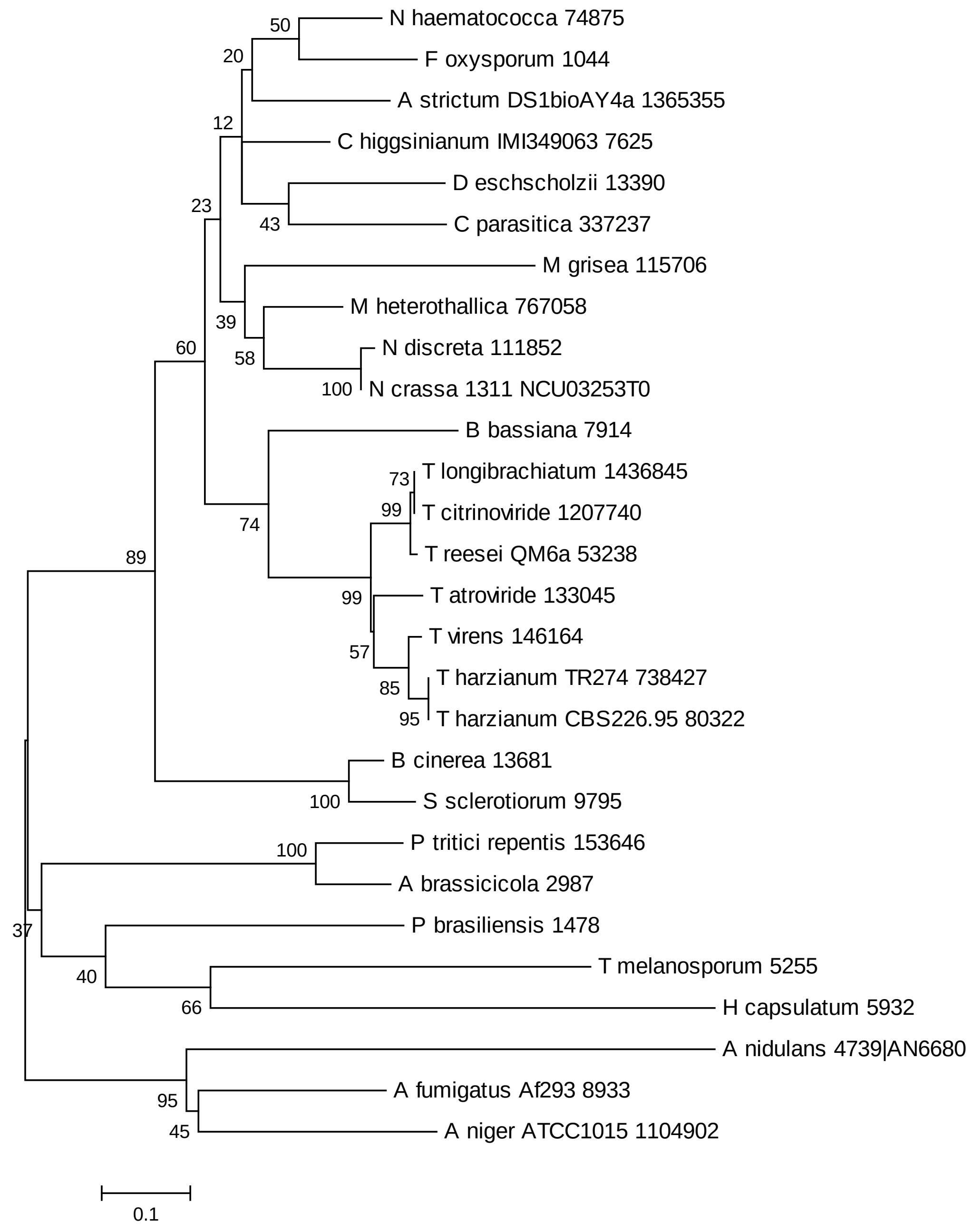

Figure S3. **Phylogenetic analysis of GPR8 (TR_53238) in fungi including human and plant pathogenic fungi.** Data were retrieved from JGI. Species names are provided along with protein IDs as listed in JGI databases. Phylogenetic analysis of protein sequences was performed with MEGA4 after sequence alignment using ClustalX. Sequences used are from *Nectria haematococca, Fusarium oxysporum, Acremonium strictum, Colletotrichum higgsinianum, Daldinia eschscholzii, Cryphonectria parasitica, Magnaporthe grisea, Myceliophthora heterothallica, Neurospora discreta, Neurospora crassa, Beauveria bassiana, Trichoderma longibrachiatum, Trichoderma citrinoviride, Trichoderma reesei, Trichoderma atroviride, Trichoderma virens, Trichoderma harzianum, Botrytis cinerea, Sclerotinia sclerotiorum, Pyrenophora tritici repentis, Alternaria brassicicola, Paracoccidioides brasiliensis, Tuber melanosporum, Histoplasma capsulatum, Aspergillus fumigatus* and *Aspergillus niger.*

Although the homologue in *A. fumigatus* is more distantly related, bidirectional best hit analysis indicates that this gene indeed encodes a homologue of *T. reesei* GPR8.

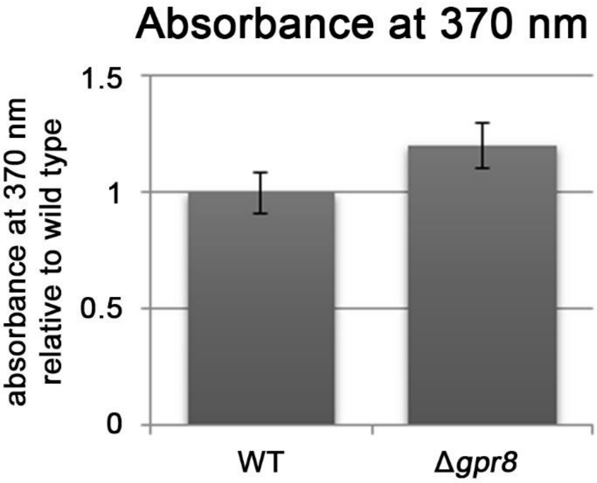

Figure S4. **Production of yellow pigments.** Deletion of *gpr8* does not significantly affect production of yellow sorbicillin derivatives as reflected by measurement of absorbance of culture filtrates at 370 nm. Errorbars reflect standard deviations of three biological replicates. Strains were grown on Mandels Andreotti minimal medium with 1 % (w/v) glucose as carbon source for 72 hours in constant darkness. Statistical evaluation did not reveal a significant difference.
