# Supplementary figures and images for: The G-protein Coupled Receptor GPR8 Regulates Secondary Metabolism in Trichoderma reesei
Source: Front Bioeng Biotechnol. 2020 Nov 5;8:558996. doi: 10.3389/fbioe.2020.558996 (PMC7676458; doi:10.3389/fbioe.2020.558996)

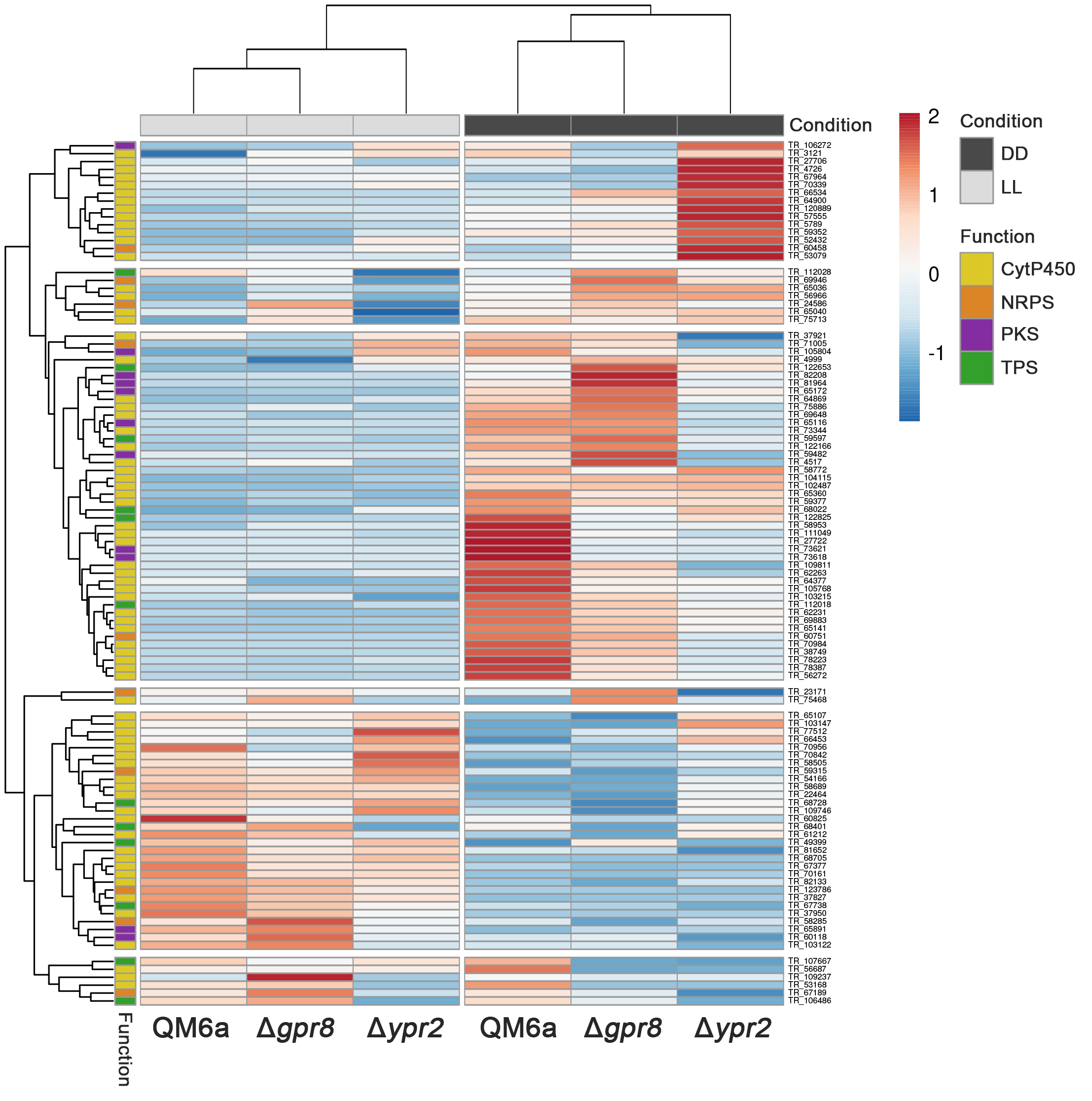

Supplement: Supplementary file 2 [file Image_1.TIF]
